# Meta-Analysis of DNA Tumor-Viral Integration Site Selection Indicates a Role for Repeats, Gene Expression and Epigenetics

**DOI:** 10.3390/cancers7040887

**Published:** 2015-11-10

**Authors:** Janet M. Doolittle-Hall, Danielle L. Cunningham Glasspoole, William T. Seaman, Jennifer Webster-Cyriaque

**Affiliations:** 1 Department of Dental Ecology, School of Dentistry, University of North Carolina at Chapel Hill, Chapel Hill, NC 27599, USA; doolittl@email.unc.edu; 2 Oral Biology Ph.D. Program, School of Dentistry, University of North Carolina at Chapel Hill, Chapel Hill, NC 27599, USA; Danielle_Cunningham@unc.edu; 3 Lineberger Comprehensive Cancer Center, School of Medicine, University of North Carolina at Chapel Hill, Chapel Hill, NC 27599, USA; Todd_Seaman@unc.edu; 4 Department of Microbiology and Immunology, School of Medicine, University of North Carolina at Chapel Hill, Chapel Hill, NC 27599, USA

**Keywords:** viral integration, HPV, HBV, MCPyV, HIV

## Abstract

Oncoviruses cause tremendous global cancer burden. For several DNA tumor viruses, human genome integration is consistently associated with cancer development. However, genomic features associated with tumor viral integration are poorly understood. We sought to define genomic determinants for 1897 loci prone to hosting human papillomavirus (HPV), hepatitis B virus (HBV) or Merkel cell polyomavirus (MCPyV). These were compared to HIV, whose enzyme-mediated integration is well understood. A comprehensive catalog of integration sites was constructed from the literature and experimentally-determined HPV integration sites. Features were scored in eight categories (genes, expression, open chromatin, histone modifications, methylation, protein binding, chromatin segmentation and repeats) and compared to random loci. Random forest models determined loci classification and feature selection. HPV and HBV integrants were not fragile site associated. MCPyV preferred integration near sensory perception genes. Unique signatures of integration-associated predictive genomic features were detected. Importantly, repeats, actively-transcribed regions and histone modifications were common tumor viral integration signatures.

## 1. Introduction

Integration into the human genome is central to transposon mutagenesis, gene therapy and viral pathogenesis [1,2,3,4]. DNA tumor virus integration has been implicated as an early oncogenic event. Viral infections are responsible for a significant portion of the global cancer burden, with over 1.2 million new cancer cases attributable to hepatitis viruses and human papillomavirus (HPV) in 2008 [5]. Integrated high-risk HPVs, principally HPV-16 and -18, are associated with anogenital and head and neck cancers (HNCs) [6]. Hepatitis B virus (HBV) integrates in up to 90% of HBV+ hepatocellular carcinomas (HCCs) [7,8]. Merkel cell polyomavirus (MCPyV) integrates in 70%–80% of Merkel cell carcinomas (MCCs), an aggressive neuroendocrine skin cancer [9,10]. Integration may increase cancer risk beyond simple infection. Following integration, increased expression of the HPV oncogenes E6 and E7 and expression of truncated forms of HBV HBx and MCPyV Tag with increased oncogenic potential have been detected [11,12,13]. In addition, viral integration can deregulate nearby human oncogenes [14,15], create oncogenic fusion genes [16,17] and contribute to genome instability [8,18]. One potential strategy for reducing cancer risk in infected or early-stage disease patients may be targeted prevention of viral integration. However, initial steps will require a better understanding of DNA tumor virus integration.

Numerous studies have been conducted to determine preferential sites of DNA tumor virus integration, with differing conclusions. HPV and HBV integrations were previously detected at common fragile sites (CFSs), prone to breakage [7,8,19,20,21,22]. HPV, HBV and HIV were thought to integrate in transcriptionally-active regions with accessible chromatin [15,23,24] and near proliferation and cancer genes, like c-Myc and hTERT [14,20,25,26,27,28,29]. Others proposed that DNA tumor virus integration occurred randomly [30,31], and cells bearing integrations selectively underwent clonal expansion and tumorigenesis, with consequent observed biases reflecting highly-represented integrations detected in cell populations [10,17,32,33]. Unlike the DNA tumor viruses, HIV encodes an enzyme to catalyze integration [34].

Large-scale analyses of integration site selection have been conducted for transposons, gene therapy vectors and HIV [1,2,3,4]. Previous analyses of tumor viral integration site preference focused on integration in CFSs or near cancer/cell growth genes [8,27,32]. However, this large-scale study is the first to compare genomic regions hosting the DNA tumor viruses HPV, HBV and MCPyV. We hypothesized that host genome properties influence tumor viral integration. We found an overall bias for integration near open chromatin regions and SINE elements for all DNA tumor viruses studied, with differences between integration sites from different virus types, cancer types and disease stages.

## 2. Results

### 2.1. Catalog of Viral Integration Sites

**Figure 1:**
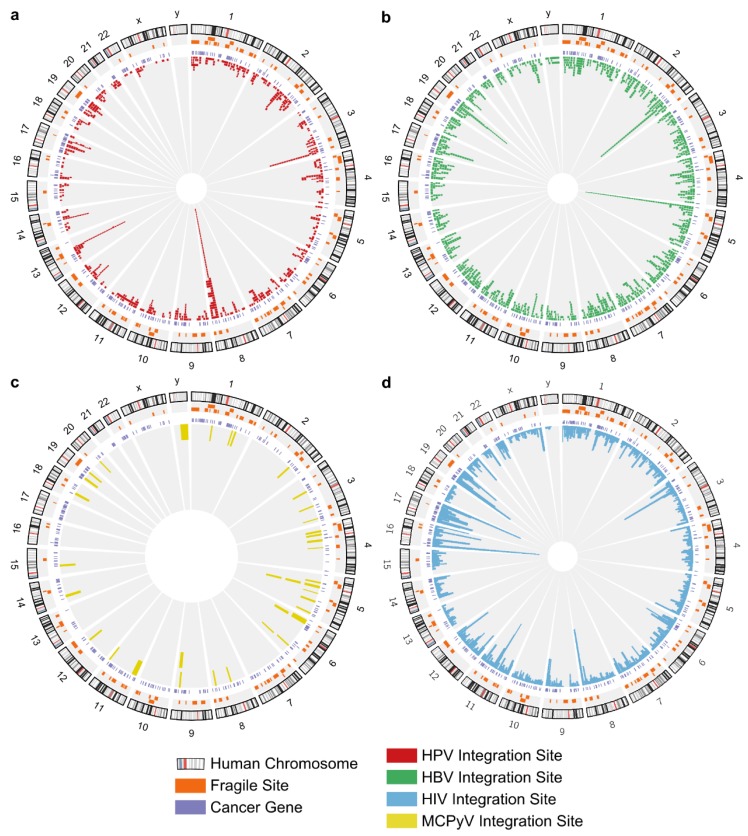
Location of viral integration sites in the human genome. Human chromosomes (1–22, X, Y) are arranged around the circle. The inner-most ring shows viral integration sites, stacking multiple events that occurred at the same location. (**a**) HPV integration sites; (**b**) HBV integration sites; (**c**) MCPyV integration sites; (**d**) HIV integration sites.

HPV, HBV, MCPyV and HIV integrations with mapped genomic positions were cataloged from the literature (references in File S1). A total of 589 HPV integration events from 436 cervical carcinomas (CESCs) and 59 HNCs were cataloged. Remaining integrations were from other carcinomas or cell lines. Several HPV types were represented, mostly HPV-16 (382) and HPV-18 (138) (Table S1, Figure S1). Some analyses below were done for the entire set of HPV integration events (*n* = 589) and for only those with precisely-mapped locations (*n* = 92). Viral integration site lengths are provided (Figure S2). HBV integration sites (1271) included HCCs (628), tumor-adjacent samples (600), both HCC and adjacent normal tissues (10) and cell lines (15) (Table S2). Thirty-seven MCPyV integration sites were identified in MCCs (34), lung cancers (two) and a cell line (one) (Table S3). HIV integration sites (45,304) were determined by Wang *et al.* by pyrosequencing [24]. Viral integration sites are shown (Figure 1), and details and references are provided (File S1, Table S4).

### 2.2. Viral Integration Hotspots

Using the z-threshold method of Presson *et al.* [35] at a 99.5 percentile threshold, multiple previously-described hotspots of HPV integration, including 8q24.11, 8q24.21 and 13q22.1, were detected [14,20,26,36]. Using our expanded catalog, 15q22.1 and 17q23.1 [19,33], which hosted multiple integration events in individual studies, were hotspots. HBV hotspots were confirmed at 5p15.33, 19q13.12, 19q13.13 (99.5 percentile threshold) and 19q12 (99 percentile threshold) [15,25,32,37] (Tables S5 and S6 and Figures S3 and S4). Interestingly, HIV had three hotspots, at 16q24.3, 11q13.1 and 6p21.33 (Table S5 and Figure S3). Additional sites are needed to assess recurrent MCPyV integration.

### 2.3. Evidence of Viral Integration Bias in Functional Genes

**Figure 2:**
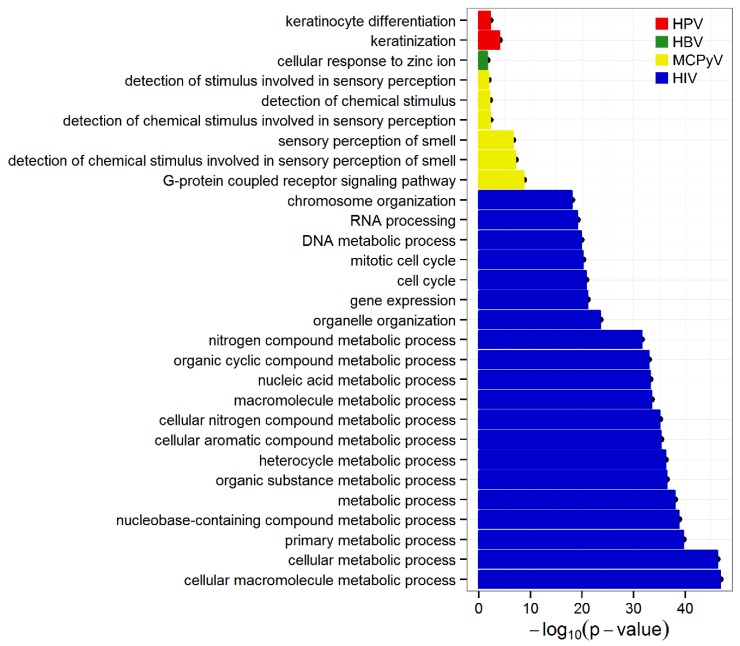
GO biological process term enrichment of genes near viral integration sites. GO terms that were significant after the Fisher exact test with Bonferroni multiple testing correction (*p* < 0.05) are shown. For HIV, only the terms with the 20 lowest *p*-values are shown.

Gene Ontology (GO) biological process terms were used to determine the integration preference near functional genes (Figure 2). Genes present within four window sizes (±100 bp, ±500 bp, ±1 kb and ±10 kb) around integration sites were assessed. For all windows, keratinocyte differentiation and keratinization terms were enriched among genes near HPV integrations. At ±10 kb, the zinc ion cellular response was enriched near HBV sites. G-protein-coupled receptor signaling and five terms associated with olfactory/sensory perception were significantly enriched for MCPyV in all windows. In agreement with a previous study [24], 196 terms related to cell cycle, mitosis, metabolism and transcription were enriched among genes within ±10 kb of HIV integrations (Figure 2). Significant GO biological process terms for the four viruses are provided (Table S7).

### 2.4. No Evidence for Preferential Integration of DNA Tumor Viruses in CFSs

Others have determined that CFSs are prone to HPV and HBV integration [7,8,19,20,21,22]. Using the binomial test, the frequency of viral integration in CFSs was compared to the fraction of the genome within CFSs. HPV, HBV and MCPyV integration rates in CFSs were not significantly different than expected (α = 0.05). However, HIV exhibited a small, but significant bias for integration in CFSs (*p* < 2.2 × 10^−16^) (Table S8). It is important to note that several integration hotspots coincide with CFSs, including HPV hotspots 8q24.1, 15q22 and 17q23.1, HBV hotspot 19q13 and HIV hotspot 11q13. However, when generalized to all integration sites, the frequency of integration in CFSs is not significantly different than expected by chance.

### 2.5. Tumor Viral Integration Did Not Require Sequence Preference

HIV and other integrase-dependent elements have preferred sequence motifs at their integration sites [1,38,39]. There are no known sequence motifs for HPV [40], HBV or MCPyV integration. Sequences ±10 bp from integration sites were examined for *de novo* motifs using HOMER, with randomly-selected sites as the background. Ten background (BG) sets with length-matched random loci for each viral integration site were selected. To control for gene density effects, ten additional random sets, gene constraint (GC) sets, were selected with a similar number of nearby genes (gene presence score). The HIV integration site motif 5′-GT(A/T)AC-3′ was recovered using both BG and GC sets. An additional motif, CGACTAGT, was identified using both random sets (Table S9). Significant motifs were not identified for HPV, HBV or MCPyV. The analysis was repeated using only precisely-mapped HPV sites. Again, no significant motifs were identified.

### 2.6. Feature Scoring and Data Integration

**Figure 3:**
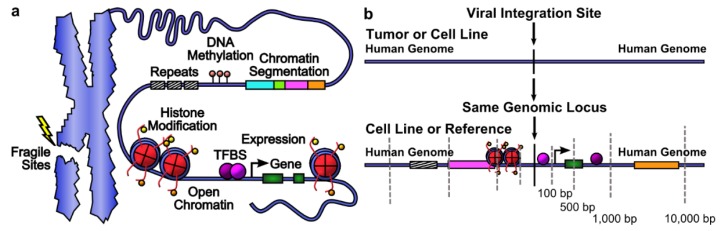
Genomic features near integration sites. (**a**) Categories of genomic features in the context of chromatin; (**b**) windows of four sizes are defined around viral integration sites, and features present in the human genome within each window are scored. Integration sites may be precisely mapped or be broader regions.

For each integration site, 277 genomic features, including gene presence, gene expression, open chromatin, histone modifications, DNA methylation, transcription factor (TF) and other protein binding, chromatin segmentations and repeats, were scored within four windows of ±100 bp, ±500 bp, ±1 kb and ±10 kb (Figure 3, Table 1, data sources in Table S10). Because gene expression, DNA-protein binding and epigenetic marks differ between cell types, cell lines were chosen that represent the viral tropism and have ENCODE data available [41]. HPV and MCPyV are epithelial-tropic, hence HPV-positive HeLa and SiHa cells and HPV-negative NHEK were used. HepG2, a hepatocyte cell line, was used for liver-tropic HBV. GM12878, a T-lymphoblastoid cell line, was used to study HIV integration [24].

### 2.7. Genomic Features Were Associated with Integration

**Figure 4:**
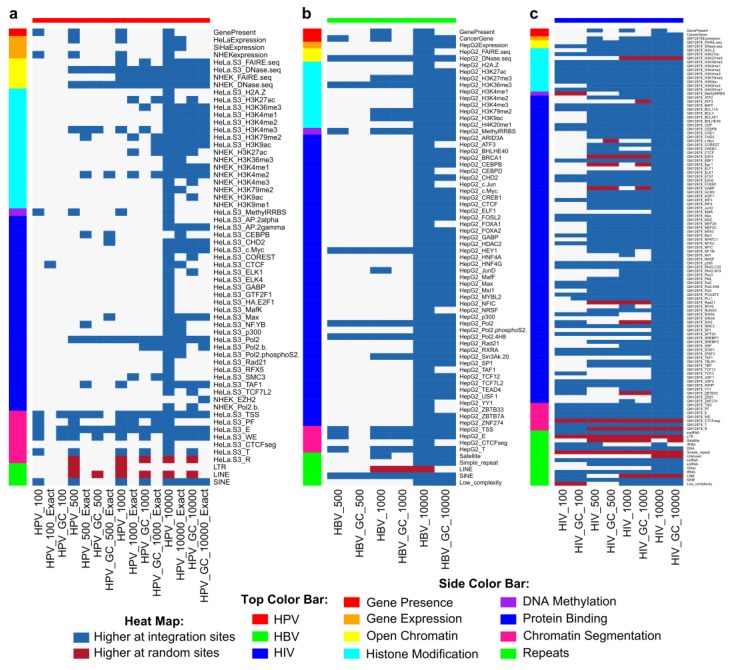
Significant differences were detected between viral integration sites and random sites. (**a**) HPV; (**b**) HBV; (**c**) HIV. Significance was determined using a two-sided Mann–Whitney U-test with Bonferroni correction, α < 0.05. Comparisons using the gene constraint set are indicated with GC. No significant differences were found for MCPyV. Only the features from the most relevant cell lines were considered for each virus.

To investigate viral integrant distribution with respect to genomic features, the Wilcoxon rank sum test with Bonferroni correction was used. Significant differences between MCPyV integration and random sites were not detected among genomic features compared to BG or GC, likely owing to low sample size. For HPV and HBV, more genomic features were significantly different in cell lines relevant to viral tropism (HPV: HeLa-S3, NHEK, SiHa; HBV: HepG2; HIV: GM12878) (Figures S5 and S6). Genomic features that did not differ between cell lines (gene presence and repeats) and genomic features from cell lines most relevant to each virus were further analyzed (HPV: 98 genomic features; HBV: 95 genomic features; HIV: 120 genomic features) (Figure 4). Most genomic features that differed scored higher at viral integration sites than random sets. However, LINE elements were significantly underrepresented, and SINE elements were preferred, near viral integration sites for these three viruses. Transcriptionally-repressed regions (R segmentation) hosted fewer HPV and HIV integrations than random. Significantly higher scores for gene presence, euchromatin, transcribed region (T) segmentation and Pol2 binding indicated that all three viruses preferred integration in gene-dense, transcribed regions. HPV showed less preference for specific TF binding than HBV or HIV. Importantly, in this study, hypermethylation of H3K4 was detected in HeLa, HepG2 and NHEK cells near integration loci for these three viruses. Methylated H3K4 has been previously associated with the introduction of double-strand DNA breaks by recombination activating gene 1 (RAG1) and RAG2 complex in lymphocytes [42].

**Table 1:** Summary of genomic features. All genomic feature scores were normalized for the length of the search region. * From ENCODE.

Category	Gene Presence	Gene Expression	Open Chromatin	Histone Modifications	DNA Methylation	TF and Other Protein Binding	Chromatin Segmentation	Repeats
Data Type	GENCODE, COSMIC Cancer Gene Census	RNA-seq	DNase-seq, FAIRE-seq	ChIP-seq	Methyl-RRBS	ChIP-seq	Hoffman *et al.* Nucleic Acids Res. 2013.	UCSC repeat masker
Data Source	hg19	HeLa *, SiHa, NHEK *, HepG2 *, GM12878 *	HeLa *, NHEK *, HepG2 *, GM12878 *	HeLa *, NHEK *, HepG2 *, GM12878 *	HeLa *, HepG2 *, GM12878 *	HeLa *, NHEK *, HepG2 *, GM12878 *	HeLa *, HepG2 *, GM12878 *	hg19
Scoring Method	Number of genes	Sum of RPKM	Number of peaks	Number of peaks	Percent Methylated	Number of peaks	Length of segment	Length of repeat
Number of Features	2	5	8	44	3	178	21	16

### 2.8. Genomic Features Predicted Integration

**Figure 5:**
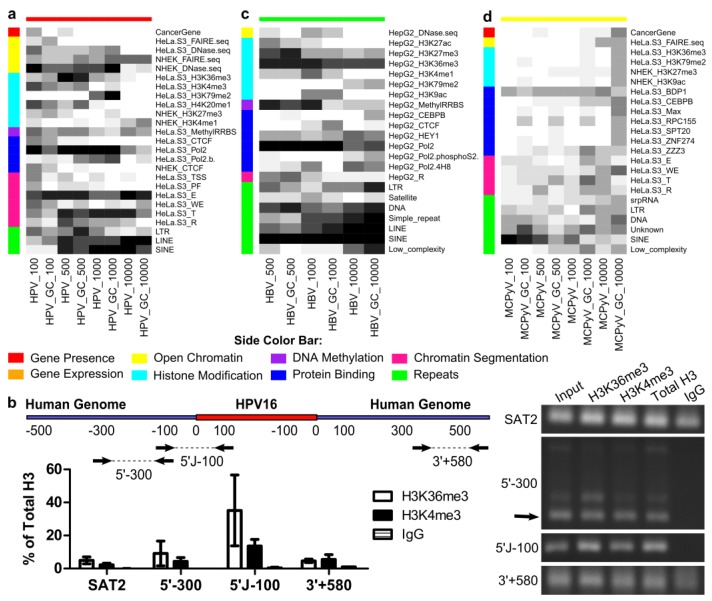
Predictive genomic features for each DNA tumor virus. Random forest models were developed for each virus and window size, using either the background set or the gene constraint set as the negative class. Starting from only the genomic features that were considered relevant to each virus, feature elimination was used to select the smallest set of features that gave an ROC within 2% of the best model using three-fold cross-validation repeated 10 times on the training set. The optimal model was then used to classify a held-out test set (75% of data for training, 25% for testing). The entire process was repeated 10 times, once for each of the randomly-selected background sets. The number of times each feature was selected for inclusion in the optimal model is shown (white: zero, black: 10). Only features selected at least five times for at least one window size are shown. (**a**) Features predictive of HPV integration. (**b**) ChIP-qPCR of two histone marks predictive of HPV integration, H3K36me3 and H3K4me3. The cartoon shows the locations of primers designed to tile across an approximately ±500-bp window around the two identical HPV-16 integrants at 13q22.1 in SiHa cells. The graph shows the mean and standard deviation of two replicates of qPCR, and a representative gel of the products is shown at the right. All primer pairs produced bands at the expected sizes, but 5′-300 showed additional bands (the arrow indicates the expected size). qPCR quantification showed high fold enrichment for 5′-300, some of which may be due to non-specific amplification. However, a band is clearly present at the expected size (arrow), suggesting the presence of H3K36me3 and H3K4me3 near the integration site. Satellite region 2 (SAT2) and total H3 were used as positive controls. (**c**) Features predictive of HPV integration. (**d**) Features predictive of HPV integration. Comparisons using the gene constraint set are indicated with GC.

Random forest (RF), a decision tree-based classification method, determined which genomic features were important for distinguishing actual DNA tumor virus integration sites from random sites. RFs were built using the 10 BG and GC sets. Genomic features associated with gene presence and repeats and cell lines most relevant to each virus were used (HPV: HeLa-S3, NHEK, SiHa (98 genomic features); HBV: HepG2 (95 genomic features); MCPyV: HeLa-S3, SiHa and NHEK (98 genomic features)). Recursive feature elimination selected the smallest subset of genomic features, resulting in an RF that performed nearly as well as the best model (Figure 5).

For HPV, RFs selected genomic features indicative of transcription (HeLa.S3_T and euchromatin), enhancers (HeLa.S3_E) and LINE and SINE elements (Figure 5a). LINEs were negative predictors of integration (Figure 4). The histone modification H3K79me2 was predictive at the ±1000-bp window, while H3K36me3, H3K4me3 and H4K20me1 were selected at smaller window sizes. Chromatin immunoprecipitation of H3K36me3 and H3K4me3 within ±500 bp of the HPV-16 integration sites in SiHa cells revealed these marks to be present in this cell line (Figure 5b). Interestingly, cancer gene presence in the immediate vicinity of integration was selected by some models based on BG sets (±100 bp, five models; ±500 bp, three models), but not when controlling for gene density (GC sets).

Within ±100 bp of HBV sites, differences from random sites were not detected (Figure 4), few genomic features were eliminated and RFs performed poorly (Table S11). For remaining window sizes, SINE elements were predictive all 10 times with both BG and GC sets (Figure 5c). LINEs were a negative predictor of integration. DNA transposon repeats and H3K36me3 were consistently selected. DNA methylation and Pol2 binding genomic features were important within shorter windows for both HBV and HPV.

Classification performance of RFs on MCPyV integration was poor (Table S11); however, some genomic features were consistently selected and warrant future investigation (Figure 5d). The most stable predictor was SINE elements within the shortest window (±100 bp), with other repeats frequently selected. Binding of BDP1, a subunit of the TFIIB complex that recruits RNA PolIII, which transcribes small ncRNAs, including SINE-encoded RNAs [43], was selected frequently at all window sizes. The largest window size had the most stable predictors.

### 2.9. HPV and HBV Integrations Differ among Classes

**Figure 6:**
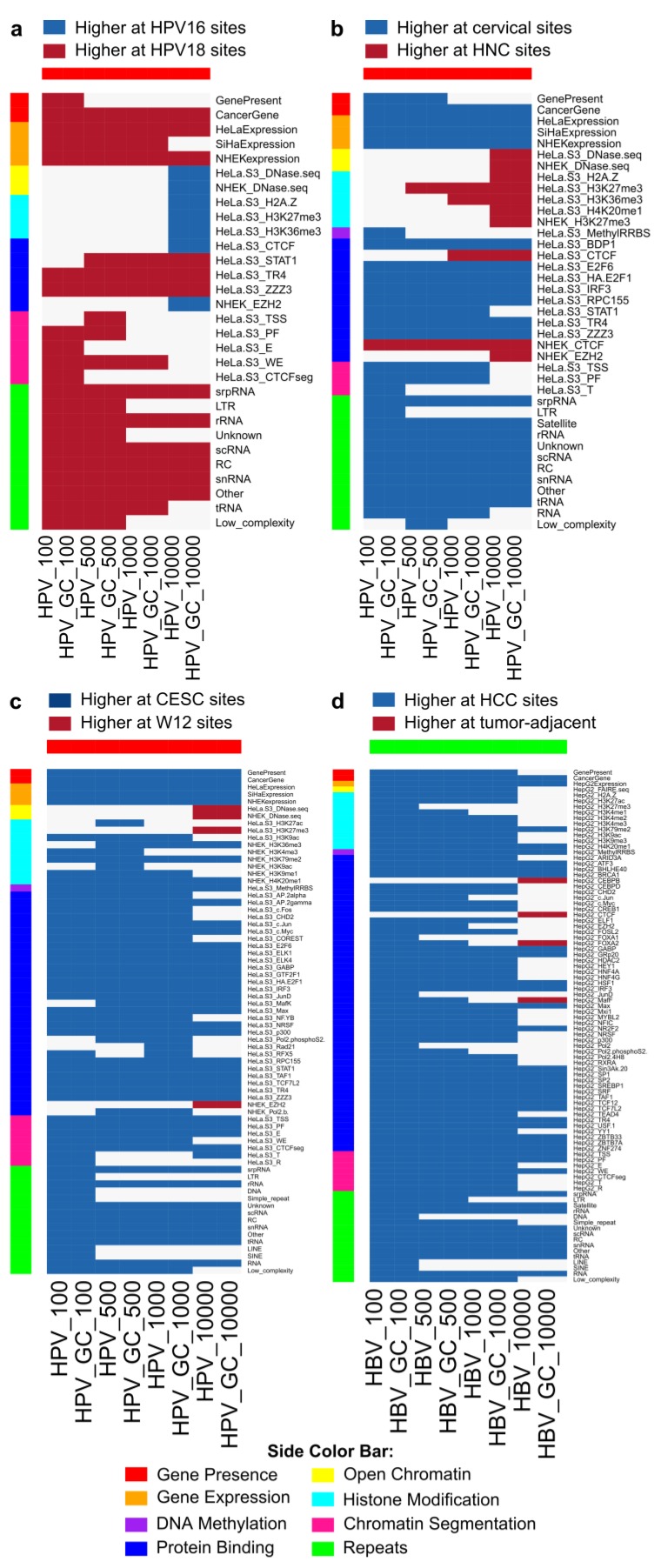
Significant differences were detected between types of viral integration sites. (**a**) Certain features in 7/8 categories were significantly different near HPV-18 integrations compared to HPV-16 (HPV-16 *n* = 382, HPV-18 *n* = 133); and (**b**) integrations in cervical tissue compared to those in head and neck cancers (HNC) (cervical *n* = 431, HNC *n* = 59). Regardless of window size or whether or not the number of genes was controlled for, gene expression, repeats and certain transcription factors differed significantly between HPV types (a) and tissues (b). (**c**) Significant differences between cervical cancer (*n* = 419) and W12 cell line (*n* = 28) integration sites. (**d**) Significant differences between HBV integration sites in HCC (*n* = 628) and tumor-adjacent tissues (*n* = 618). Significance was determined using a two-sided Mann–Whitney U-test with Bonferroni correction, α < 0.05. Comparisons using the gene constraint set are indicated with GC.

HPV integration events represented several cancer and HPV types. To investigate potential differences in integration site preferences between subsets, differences in genomic features were determined by the Mann–Whitney U-test with Bonferroni correction. Ninety-eight genomic features based on gene presence and repeats sequence elements and genomic features from HeLa, SiHa and NHEK cells were used (Figure 6).

HPV types 16 and 18 are the most prevalent in cancer. Most genomic features that differed between HPV-16 and HPV-18 integration sites scored higher at HPV-18 sites (24/31 genomic features). Open chromatin, histone modifications, CTCF binding and EZH2 were higher at HPV-16 sites for the ±10-kb window (Figure 6a). Genomic features associated with HPV-18 sites were less sensitive to window size.

Comparing integrations in cervical tissues and HNCs (Figure 6b), gene presence, gene expression, DNA methylation, TF binding, chromatin segments and repeats differed significantly and scored higher near cervical tissue integrations. Euchromatin regions, histone modifications, CTCF and EZH2 binding were higher near HNC integration events.

Events surrounding spontaneous integration in the W12 cell line were compared to integration events in established CESC. Clonal expansion of cells bearing integrations conferring a selective advantage has been observed [10,17,32,33,44,45], so selectively disadvantageous or neutral integrations could be underrepresented in tumor biopsies. Lacking *in vivo* selective pressures, W12 integrations may better represent the integration process than integration sites observed in cancers (Figure 6c) [46]. Over two-thirds of genomic features were significantly different between CESC and spontaneous W12 integrations (59/98). Preference for CESC integration near cancer genes and higher gene expression were detected at all window sizes in cancers compared to W12. At the largest window size, few genomic features were found more often near W12 integration loci, including euchromatin-associated DNase hypersensitive regions and epigenetic indicators of transcriptionally-silent regions: H3K27me3 (HeLa-S3) and EZH2 binding (NHEK). In NHEK cells, histone marks associated with active transcription (H3K36me3, H3K4me3 and H3K9ac) were detected at loci that hosted integrations in cancers. Only one repressive mark was detected, H3K9me1, indicating that these regions are generally open. H3K79me2 and H4K20me1, both associated with DNA replication and maintenance of genomic stability [47,48], were frequently detected at cancer integration sites. In HeLa-S3, several TFs were bound near integration loci from cancer cases (ENCODE data not available for NHEK). However, HeLa-S3 data cannot distinguish whether gene expression and epigenetic genomic features were present before integration or reflect changes accumulated during cancer progression.

Previous studies detected differences between HBV integration sites in non-tumor and tumor tissues, possibly due to clonal selection and oncogenesis [15,32]. For HBV, 95 genomic features from sequence elements and HepG2 cells were analyzed. Comparing HBV integrations in HCC tumor samples and tumor-adjacent tissues, 91 of 95 genomic features were significantly different after Bonferroni correction, most of which scored higher near tumor integrations (Figure 6d). Exceptions were detected at the longest range analyzed and included CEBP, CTCF, FOXA2 and MafF binding.

## 3. Discussion

Integration is critical to the pathogenesis of multiple viruses. In this large-scale study, 277 genomic features were assessed around known DNA tumor virus integration sites. DNA tumor virus integrants were not associated with consensus sequence motifs, and repeat regions predicted their integration. Unlike previous studies, comparisons to random chance indicated that HPV and HBV have no preference for CFSs, although HIV may. While integration hotspots 8q24.1, 15q22, 17q23.1, 19q13 and 11q13 were within CFSs, this meta-analysis did not detect integration events at higher frequency in CFSs compared to elsewhere in the genome. Integration occurred near features suggesting viral tropism: GO term enrichment in keratinization for HPV, sensory perception for MCPyV and cell type-specific genomic features. Viral preference for transcriptionally-active gene-dense regions and accessible chromatin was confirmed [15,23,24]. Interestingly, epigenetic modifiers were consistently associated with all viral integration events, and specific integration-associated marks included H3K36me3, H3K4me3, Pol2 binding and DNA methylation. Of interest, methylated CpGs within fragile zones of oncogenes prone to translocation events are targeted by the AID enzyme, resulting in double-stranded DNA breaks [49], which could facilitate an integration event.

Previous studies noted hotspots of HPV integration in 8q24 and 13q22.1 [14,20,26,36]. While common high-risk HPVs frequently integrate in 8q24, our results suggest HPV-18 has a stronger preference than HPV-16. Repeats, enhancers, Pol2 binding, open chromatin and histone modifications (H3K36me3, H3K4me3, H3K79me2 and H4K20me1) predicted HPV integration. These histone marks indicate DNA damage, replication and maintenance of genomic stability [47,48], suggesting integration in damage-prone regions even in the absence of evidence of preference for CFSs. Viral infection has also been tightly associated with initiation of APOBEC enzymatic machinery, which causes double-strand DNA breaks that may facilitate integration [50]. DNA replication may provide open regions for viral integration, and disruption of these marks may contribute to the frequently-observed genomic instability near HPV integration.

The state of genomic features at the time of integration and within a particular cell is uncertain. The determination of genomic features using extensive genome-wide studies across biopsies was impractical, and widely studied cell line data provided reasonable models. HeLa, SiHa and NHEK originate from anogenital epithelium; however, this may differ from oral epithelium present in HNCs. NHEK approximated the epithelial cell epigenome prior to virus integration. W12 integration loci should reflect early integration events, while HeLa cells likely reflect accumulated epigenetic cancer-associated changes post-integration.

Genomic features present near integration loci in HPV+ cancers differed from those near W12 integration loci. Likewise, clear differences were detected between HBV integrations in tumor and adjacent non-tumor sources. These data support the hypothesis that only some HPV and HBV integrations lead to functional changes promoting tumorigenesis. Studies of integration in matched tumor and normal tissues are lacking for HPV, and further investigation is warranted. While comparisons may be confounded by the abundance of HPV-16+ HNC cases, integrants demonstrated clear differences in the integration site genomic feature profile of HPV-16 *vs.* HPV-18 and HNC *vs.* cervical tissues. More features were statistically associated with HPV-18 and cervical integrations.

HBV integration sites frequently occurred in cancer-related genes, and integration may deregulate these genes in HCC. Preferential integration into actively-transcribed genes may reflect open chromatin configuration [27], and integration into hTERT, MLL4/KMT2B, CCNE1 and others have been reported [15,25,27,29,32,37,51,52]. Recent studies detected integration in promoters, exons and cancer-related genes more frequently in tumor samples than tumor-adjacent tissue [15,27]. However, others concluded there was no difference in integration near cancer-related genes [8]. Like the former, we detected a preference for HBV integration in gene-dense regions and cancer genes in HCC. We found no association with HBV integrations in CFSs.

MCPyV, integrated in 70%–80% of Merkel cell carcinomas [9,10], is newly discovered with few mapped integration loci. Neuroendocrine tumors displayed an interesting association between MCPyV integration and sensory perception and G-protein-coupled receptor genes. MCPyV integration occurred preferentially near SINEs and BDP1 binding sties. Identification of additional MCPyV integration sites is needed for confirmation.

Unlike the tumor viruses, HIV demonstrated a bias for CFSs and sequence motifs. Previous studies of HIV integration in GM12878 cells covered 1% of the genome [24]. Our analysis covered the entire genome, confirming that HIV integrates less often in LINEs and more often in metabolism, cell cycle and mitosis-associated genes [24]. An association between HIV integration sites and transcriptionally-active epigenetic marks was noted [24]. Our results align with previous studies, validating our methods.

Determining DNA tumor virus integration site preference aids the understanding of virus-mediated tumorigenesis. Unlike HIV, for which effective HIV integrase inhibitors exist [34], viral and human proteins were previously not known to be essential for HPV, HBV or MCPyV integration. Understanding features that make human genomic loci prone to DNA tumor virus integration is an important first step to unveil druggable targets to prevent integration in infected patients.

## 4. Materials and Methods

### 4.1. Detection of HPV-16 Integration in Oral Cancer

DIPS-PCR was performed on 5 oral cancer biopsies according to Luft *et al.* [31]. PCR products were purified and sequenced. BLAST analysis was performed to determine homology with HPV-16 and the human genome [53]. The study was conducted in accordance with the Declaration of Helsinki, and the protocol was approved by the Institutional Review Board of the University of North Carolina at Chapel Hill (IRB# 05-DENT-1263-ORC).

### 4.2. Catalog of Viral Integration Sites

In addition to the five novel oral cancer HPV integration sites identified by DIPS-PCR, HPV, HBV, MCPyV and HIV integrations with mapped genomic positions were cataloged from the literature (references in File S1). For HPV, depending on the methods, study focus and knowledge at the time, some integration sites were previously reported as “in” or “near” a gene or CFS. For HPV, HBV and MCPyV, some studies did not report precise genomic locations. Reported sequences were mapped to the human genome using BLAST [53]. When no sequence was provided, the cytoband that contains the nearby gene or CFS was taken to be the integration site, termed a broad site. Analyses were done for the entire set of HPV integration events (*n* = 589) and for only those with precisely-mapped locations (*n* = 92). Viral integration site lengths are provided (Figure S2). HIV integration site sequences [24] were retrieved from GenBank (EI522403–EI666579) and mapped to hg19 using the BLAST-like Alignment Tool (BLAT) [54]. Hits were filtering to remove matches that started further than 3 nucleotides from the HIV LTR, as in the original analysis, resulting in 45,304 HIV integration sites. Details of integration sites and references are provided (File S1, Table S4). Circos plots were used to visualize viral integration sites in the human genome [55].

### 4.3. Hotspots

Hotspots of viral integration were determined using a method adapted from the z-score threshold method of Presson *et al.* [35]. Briefly, the integration sites in each cytoband were counted (if a site crossed more than cytoband, each was given an equal fractional count) and divided by the length of the cytoband in Mb before calculating z-scores using R 3.0.2 [56]. Because viral integration counts were adjusted for cytoband length, integration events falling within a short cytoband could be considered a hotspot. The threshold was set at the 99.5 percentile (*z* = 210.31) or 99 percentile (*z* = 79.02) of HPV z-scores.

### 4.4. GO Term Enrichment Analysis

GO biological process terms were associated with human genes using biomaRt 2.22.0 [57,58]. GO terms enriched among the genes found within windows around viral integration sites were identified using the R package topGO 2.18.0 [59] with all human genes as the background set [59]. Significantly-enriched terms were found using the Fisher exact test with Bonferroni multiple testing correction (α = 0.05).

### 4.5. Fragile Sites

The binomial test (binom.test, R 3.0.2 [56]) was used to compare the frequency of integration sites in CFSs to the fraction of the genome that falls within CFSs. CFSs were defined according to [60].

### 4.6. Random Sites

Viral integration sites were compared to 10 sets of randomly-selected genomic loci, called the background (BG) set. For integration sites with inexact mapping, the exact randomly-selected position was used for determining the background frequency of integration in a CFS and expanded symmetrically to make a region of the same length as the actual integration site for genomic feature scoring. For HPV, the Y chromosome was excluded, because no real integration sites were observed on Y. A second group of background sets was selected for each virus and window size where each integration site was matched to 10 random loci having a gene presence score (genomic feature scoring, below) within ±5% of the actual site’s score. This random set is referred to as the gene constraint (GC) set.

### 4.7. Motif Finding

The sequences 10 bp upstream and downstream of viral integration sites or random sites were retrieved from the human genome using SAMtools 0.1.19 [61]. HOMER v. 4.6 [62] (http://homer.salk.edu/homer/motif/) was used for *de novo* motif discovery among the sequences around the integration sites, with either the sequences around the BG set or the GC set used as the background sequences. Motifs with a *p*-value of <1 × 10^−10^ and present in at least 5% of the target sequences were considered significant.

### 4.8. Genomic Feature Scoring

Genomic features were scored within windows of ±100 bp, ±500 bp, ±1 kb and ±10 kb from the integration site. Depending on the method used, some integration sites from the literature were specified as a single nucleotide, while others were reported only as an approximate region. Integration sites specified as a single nucleotide have the smallest genomic region after the addition of the surrounding window, defined as the unit region. Feature scores were normalized by the number of unit regions in the genomic region, after subtracting the length of gaps in the reference assembly.

Two hundred seventy seven genomic features, divided into 8 categories, were scored as follows: Category 1 consisted of two features, scored as the number of genes present, according to GENCODE [63], and the number of genes linked to cancer, defined by the COSMIC Cancer Gene Census [64]. Category 2, gene expression, was comprised of 5 features. Four were RNA-seq of HeLa-S3, NHEK, HepG2 and GM12878 from ENCODE [41]. HeLa-S3 are cervical epithelial cells with HPV-18 integration, and NHEK is an HPV-negative anogenital epithelium. HepG2, a hepatocyte cell line, was used for liver-tropic HBV. GM12878 is a T-lymphoblastoid cell line previously used to study HIV integration site selection [24]. Additionally, we performed RNA-seq on SiHa cells, using Illumina Hi-Seq 2000 with 100-bp single-end reads. All RNA-seq data were processed in the same way, by mapping to hg19 using TopHat v. 2.0.9 and calculating RPKM with CuffLinks v. 2.1.1. Replicates were averaged. The expression levels of genes in the window was summed. SiHa RNA-seq data are available at GEO (GSE67115). Category 3 pertains to open chromatin regions and consists of 8 features from ENCODE, DNase-seq and FAIRE-seq on HeLa-S3, NHEK, HepG2 and GM12878. Category 4 included 44 histone modifications measured by ENCODE using ChIP-seq in the four cell lines. Category 5 was methyl-RRBS data from ENCODE, measuring the percent DNA methylation in each window for the four cell lines. Category 6 includes 178 features for TF and protein binding from ENCODE data on HeLa-S3, NHEK, HepG2 and GM12878 cells. Scores for Categories 3, 4 and 6 were the counts of peaks that fall completely within the windows. Category 7 was the 7 chromatin segmentation states determined by Hoffman *et al.* using ChromHMM and Segway on HeLa-S3, HepG2 and GM12878 [65]. These seven states are based on histone modifications, Pol2, CTCF and open chromatin and include transcription start site (TSS), promoter flanking (PF), enhancer (E), weak enhancer (WE), CTCF binding (CTCF), transcribed region (T) and repressed or inactive region (R) [65]. As stated above, all feature scores were normalized for the length of the region. A full list of features and associated data sources is given in Table S10.

### 4.9. Statistical Analysis

To determine whether genomic features detected around loci differ between HPV types, cancer types, cancer *vs.* normal samples or actual integrations sites *vs.* random sites, the Mann–Whitney U-test was performed. The Bonferroni method was used to correct for multiple testing, with α = 0.05. The results were visualized in a heatmap, using the heatmap.2 function in the gplots 2.16.0 R package [66], without clustering.

### 4.10. Selection of Features to Classify Integration and Random Sites

RF models were developed for each virus and window size, using either the BG set or GC set as the negative class. For each of the 10 sets of each negative class, the data were split into a held-out testing set (25%) and a training set (75%). Using three-fold cross-validation repeated 10 times, feature elimination was used to select the smallest set of features that gave an ROC within 2% of the best model using the R package caret 6.0–30 and rfe function [67]. For each fold, the features were selected using 2/3 of the training data, and the remaining 1/3 was used to calculate ROC. The number of times (max = 10) each genomic feature was selected for inclusion in the final model using each random set was counted. The optimal model was then used to classify the remaining 25% of the data that was held-out of the entire feature selection and training process (testing set), and the accuracy, sensitivity and specificity of classification of the test set were averaged over the 10 RFs (Table S11). Genomic features that were selected in most RFs (Figure 5) and all selected genomic features are shown (Figure S7). The features selected for each RF were visualized using the heatmap.2 function in the gplots 2.16.0 R package [66], without clustering.

### 4.11. Cell Culture

SiHa cells (ATCC HTB-35) were grown in 5% CO_2_ in High Glucose Dulbecco’s Modified Eagle Medium (DMEM) (Gibco) supplemented with 10% fetal bovine serum (FBS) (Gibco).

### 4.12. Chromatin Immunoprecipitation Assay

SiHa cells were seeded (5 × 10^6^ cells) on 15-cm dishes. At 90% confluence, cells were treated with 37% formaldehyde (Sigma) added to a final concentration of 1% and incubated for 10 min. Glycine was added to cells at 10× for 5 min to quench crosslinking. Both fixation and quenching steps were performed at room temperature and with constant rotation in a fume hood. Chromatin preparation: nuclei preparation, chromatin shearing and subsequent immunoprecipitations were performed using the Simple ChIP Plus Enzymatic Chromatin IP Kit (Cell Signaling Technology, Danvers, MA, USA) with the following modifications. Chromatin was enzymatically digested by the addition of 2 μL micrococcal nuclease and incubation at 37 °C for 15 min. Chromatin digest was stopped by the addition of 40 μL of 0.5 M EDTA. DNA concentration was obtained from 25-μL samples, and equal amounts of chromatin were added to each immunoprecipitation (IP) based on these calculations. Immunoprecipitation: 2% of the chromatin from each treatment condition was stored as the input control. Four micrograms of chromatin per IP were used. Lysates were pre-cleared for 1 h with rotation using protein-G magnetic beads (Cell Signaling Technology), then incubated overnight at 4 °C with either: (1) anti-histone H3 lysine 4 tri-methyl (H3K4me3) (EpiCypher Inc., Chapel Hill, NC, USA) at a concentration of 2 μL/IP; (2) anti-histone H3 lysine 36 tri-methyl (H3K36me3) at a concentration of 2 μL/IP (Cell Signaling Technology); (3) anti-histone H3 XP (Cell Signaling Technology) at 10 μL/IP; or (4) normal rabbit IgG (negative control) (Cell Signaling Technology) at 1 μL/IP of chromatin. Ten microliters of protein-G magnetic beads were added to each IP, and the incubation was continued for 2 h at 4 °C. Bead-chromatin complexes were washed and eluted, and cross-linking was reversed using 5 M NaCl and proteinase K. DNA was purified using either the provided purification spin columns or the ChIP DNA Clean & Concentrator kit (Zymo Research). Precipitated DNA was diluted 1:2 in dH_2_O and used for real-time qPCR.

### 4.13. Chromatin Immunoprecipitation Quantification via qPCR

Real-time qPCR was performed using the Roche Lightcycler 480 (Roche Diagnostics, Indianapolis, IN, USA) using the following protocol: 95 °C for 10 min, followed by 45 cycles of 95 °C for 15 s, 60 °C (or 53 °C) for 1 min followed by signal acquisition. PCR reactions were assembled in a PCR hood using 1× SYBR Green PCR Master Mix (Roche Diagnostics), 300 nM primers and 2 μL of diluted template for a total reaction volume of 10 μL. Primers were chosen to approximate a ±500-bp window around the two HPV-16 integration sites at 13q22.1 in SiHa cells. The two integrated copies have identical breakpoints and are separated by a duplicated segment of the human genome [18]. Primer sets covering two regions failed to produce an amplification product: the region from the 3′ junction to approximately 400 bp 3′ and approximately 300–500 bp 5′ of the 5′ junction, so histone marks in these sections of the window were not observed. Primer sequences are given in Table S12. To confirm qPCR signal and amplification, the qPCR reactions were diluted 1:2 and run on a 3% TBE-agarose gel stained with 0.05 μg/mL ethidium bromide. Fold enrichment was determined as follows: input dilution factor (2%) = log_2_(50) = 5.64; ∆Ct input normalized IP value = (Ct[IP] − Ct[input × 5.64]); IgG adjusted IP Ct value (∆∆Ct) = (∆Ct[IP] − ∆Ct[IgG]); fold enrichment above IgG = 2^(−∆∆Ct)^.

## Supplementary Materials

Supplementary materials can be accessed at: http://www.mdpi.com/2072-6694/7/4/0887/s1. File S1. Supplemental references, tables and figures. Contains Tables S1–S3, S5, S6, S8, S11, and S12 and Figures S1–S7. Table S4: Viral integration sites characterized in this study. Source: reference for integration site; cellType: cell or tissue in which integration site was found; chrBand: cytoband in which the integration site falls; Coordinates: genomic locus of integration; FusionTranscript: whether a viral-human fusion transcript was detected; Sample Name: sample id used in the original study; Accession: identifier for nearest reported gene to integration site; CoorMethod: method used to determine the integration coordinates; ExpMethod: method used to determine the integration site in the original study; NuclAssayed: type of nucleic acid assayed by the experimental method. Table S7: GO terms that were significantly enriched within the windows around viral integration sites. Table S9: Motifs enriched at HIV integration sites compared to the random loci (BG or GC sets). Table S10: Details of data sources for each genomic feature.
