# Supplementary material for: Meta-Analysis of DNA Tumor-Viral Integration Site Selection Indicates a Role for Repeats, Gene Expression and Epigenetics
Source: Cancers (Basel). 2015 Nov 10;7(4):2217–35. doi: 10.3390/cancers7040887 (PMC4695887; doi:10.3390/cancers7040887)
Supplement: Supplementary file 1 [file cancers-07-00887-s001.zip › cancers-87069-supplementary - final/File_S1.docx]

**Supplementary Information**

**Viral Integration Sites**

Viral integration sites were identified in the literature for HPV [1–25], HBV [8,9,26–59], MCPyV [60–66] and HIV [67].

**Table S1.** HPV type and origin for cases with known integration site locations.

| **HPV type** | **Cervical** | **HNC** | **Cell Line** | **Vaginal** | **Vulvar** | **Penile** | **Lung** | **Renal** | **Bladder** | **Total** |
| --- | --- | --- | --- | --- | --- | --- | --- | --- | --- | --- |
| HPV16 | 247 | 51 | 72 | 3 | 5 | 3 | 1 |  |  | **382** |
| HPV18 | 129 | 1 | 6 | 1 |  |  |  | 2 |  | **138** |
| HPV18-related | 1 |  |  |  |  |  |  |  |  | **1** |
| HPV26 |  | 1 |  |  |  |  |  |  |  | **1** |
| HPV31 | 6 |  |  |  |  |  |  |  |  | **6** |
| HPV33 | 8 | 5 |  |  |  |  |  |  |  | **13** |
| HPV39 | 1 |  |  |  |  |  |  |  |  | **1** |
| HPV45 | 31 |  |  |  |  |  |  |  | 1 | **32** |
| HPV52 | 4 |  |  |  |  |  |  |  |  | **4** |
| HPV56 | 2 | 1 |  |  |  |  |  |  | 1 | **4** |
| HPV58 | 3 |  |  |  |  |  |  |  |  | **3** |
| HPV59 | 1 |  |  |  |  |  |  |  |  | **1** |
| HPV68b | 1 |  |  |  |  |  |  |  |  | **1** |
| HPV69 | 1 |  |  |  |  |  |  |  |  | **1** |
| HPV70 | 1 |  |  |  |  |  |  |  |  | **1** |
| Total | **436** | **59** | **78** | **4** | **5** | **3** | **1** | **2** | **2** | ***589*** |

**Table S2.** HBV origin for cases with known integration site locations.

| **HCC** | **HCC and Adjacent Tissue** | **Non-Tumor** | **Cell Line** | **Total** |
| --- | --- | --- | --- | --- |
| 628 | 10 | 618 | 15 | ***1271*** |

**Table S3.** MCPyV origin for cases with known integration site locations.

| **MCC** | **Lung Cancer** | **Cell Line** | **Total** |
| --- | --- | --- | --- |
| 34 | 2 | 1 | ***37*** |

**Table S5.** Viral integration hotspots at a 99.5 percentile threshold.

| **Virus** | **Cytoband** | **Raw Count** | **Normalized Count** | **z-score** |
| --- | --- | --- | --- | --- |
| **HPV** | 8q24.11 | 8.5 | 5.67 | 218.48 |
| **HPV** | 8q24.21 | 28.5 | 6.79 | 263.43 |
| **HPV** | 13q22.1 | 19.5 | 9.29 | 363.86 |
| **HPV** | 15q22.1 | 2 | 10.00 | 392.55 |
| **HPV** | 17q23.1 | 6 | 8.57 | 335.16 |
| **HPV16** | 13q22.1 | 13.5 | 6.43 | 360.43 |
| **HPV16** | 15q22.1 | 2 | 10.00 | 565.24 |
| **HPV18** | 8q24.11 | 4.5 | 3.00 | 271.57 |
| **HPV18** | 8q24.21 | 11.5 | 2.74 | 247.38 |
| **HPV18** | 17q23.1 | 5 | 7.14 | 654.13 |
| **HBV** | 5p15.33 | 42.5 | 9.44 | 400.55 |
| **HBV** | 19q13.12 | 16 | 5.71 | 235.03 |
| **HBV** | 19q13.13 | 4 | 10.00 | 425.20 |
| **MCPyV** | 3q27.1 | 1 | 0.56 | 278.98 |

**Table S5.** *Cont*.

| **Virus** | **Cytoband** | **Raw Count** | **Normalized Count** | **z-score** |
| --- | --- | --- | --- | --- |
| **MCPyV** | 4q22.2 | 1 | 0.71 | 360.17 |
| **MCPyV** | 5q14.2 | 1 | 0.71 | 360.17 |
| **HIV** | 6p21.33 | 302 | 177.65 | 223.69 |
| **HIV** | 11q13.1 | 484.5 | 193.80 | 246.00 |
| **HIV** | 16q24.3 | 322 | 194.59 | 247.09 |

**Table S6.** Viral integration hotspots at a 99 percentile threshold. * Cytobands suggested to be hotspots of MCPyV integration due to a small number of known integrations.

| **Virus** | **Cytoband** | **Raw Count** | **Normalized Count** | **z-score** |
| --- | --- | --- | --- | --- |
| HPV | 3q28 | 19 | 4.32 | 164.31 |
| HPV | 4q21.3 | 5.5 | 5.00 | 191.70 |
| HPV | 8q24.11 | 8.5 | 5.67 | 218.48 |
| HPV | 8q24.21 | 28.5 | 6.79 | 263.43 |
| HPV | 13q22.1 | 19.5 | 9.29 | 363.86 |
| HPV | 14q24.1 | 11 | 4.78 | 182.96 |
| HPV | 15q22.1 | 2 | 10.00 | 392.55 |
| HPV | 17q23.1 | 6 | 8.57 | 335.16 |
| HPV | 3p21.33 | 1 | 2.50 | 91.27 |
| HPV16 | 1p36.33 | 3.5 | 1.52 | 79.04 |
| HPV16 | 3q28 | 14 | 3.18 | 174.24 |
| HPV16 | 4q21.3 | 4 | 3.64 | 200.31 |
| HPV16 | 8q24.11 | 4 | 2.67 | 144.70 |
| HPV16 | 8q24.21 | 15 | 3.57 | 196.58 |
| HPV16 | 12q14.3 | 5 | 1.92 | 102.06 |
| HPV16 | 13q22.1 | 13.5 | 6.43 | 360.43 |
| HPV16 | 14q24.1 | 5 | 2.17 | 116.44 |
| HPV16 | 15q22.1 | 2 | 10.00 | 565.24 |
| HPV18 | 1q42.11 | 0.5 | 1.00 | 86.89 |
| HPV18 | 3p21.33 | 0.5 | 1.25 | 109.97 |
| HPV18 | 3q25.2 | 4 | 1.38 | 121.91 |
| HPV18 | 4q21.3 | 1.5 | 1.36 | 120.46 |
| HPV18 | 8p11.23 | 2 | 1.11 | 97.15 |
| HPV18 | 8q24.11 | 4.5 | 3.00 | 271.57 |
| HPV18 | 8q24.21 | 11.5 | 2.74 | 247.38 |
| HPV18 | 13q22.1 | 3 | 1.43 | 126.46 |
| HPV18 | 17q23.1 | 5 | 7.14 | 654.13 |
| HPV18 | 17q23.3 | 2 | 1.33 | 117.67 |
| HBV | 10q11.21 | 9 | 2.37 | 86.56 |
| HBV | 16q24.3 | 4 | 2.42 | 88.72 |
| HBV | 19q13.12 | 16 | 5.71 | 235.03 |
| HBV | 19q13.13 | 4 | 10.00 | 425.20 |
| HBV | 2q33.2 | 5 | 3.13 | 120.13 |
| HBV | 2q35 | 30.5 | 4.92 | 199.75 |
| HBV | 4q33 | 4 | 2.22 | 80.07 |
| HBV | 5p15.33 | 42.5 | 9.44 | 400.55 |
| HBV | 8q11.22 | 1 | 2.50 | 92.39 |
| MCPyV | 1p22.3 | 1 | 0.29 | 140.96 |
| MCPyV | 1q25.1 | 1 | 0.32 | 159.82 |

**Table S6.** *Cont*.

| **Virus** | **Cytoband** | **Raw Count** | **Normalized Count** | **z-score** |
| --- | --- | --- | --- | --- |
| MCPyV | 1q31.1 | 1 | 0.20 | 97.12 |
| MCPyV | 2q32.3 | 1 | 0.18 | 87.82 |
| MCPyV | 3p14.2 | 1 | 0.20 | 95.11 |
| MCPyV | 3q21.3 | 1 | 0.29 | 145.26 |
| MCPyV | 3q27.1 | 1 | 0.56 | 278.98 |
| MCPyV | 4p14 | 1 | 0.19 | 89.54 |
| MCPyV | 4q22.2 | 1 | 0.71 | 360.17 |
| MCPyV | 5p13.2 | 1 | 0.22 | 106.01 |
| MCPyV | 5q11.2 | 3 | 0.37 | 181.95 |
| MCPyV | 5q14.2 | 1 | 0.71 | 360.17 |
| MCPyV | 5q35.1 | 1 | 0.23 | 113.77 |
| MCPyV | 6p22.3 | 2 | 0.20 | 97.12 |
| MCPyV | 6p24.1 | 0.5 | 0.28 | 136.90 |
| MCPyV | 6q23.3 | 1 | 0.26 | 129.42 |
| MCPyV | 6q24.1 | 1 | 0.26 | 129.42 |
| MCPyV | 8q12.3 | 1 | 0.26 | 129.42 |
| MCPyV | 8q24.21 | 1 | 0.24 | 116.60 |
| MCPyV | 9q33.1 | 1.5 | 0.31 | 154.66 |
| MCPyV | 11q25 | 1 | 0.24 | 116.41 |
| MCPyV | 12q23.1 | 1 | 0.19 | 89.54 |
| MCPyV | 19q13.2 | 1 | 0.21 | 103.65 |
| MCPyV | 20q11.21 | 1 | 0.37 | 184.26 |
| HIV | 1q22 | 177 | 118.00 | 141.31 |
| HIV | 3p21.31 | 550 | 85.94 | 97.04 |
| HIV | 6p21.32 | 140 | 100.00 | 116.46 |
| HIV | 6p21.33 | 302 | 177.65 | 223.69 |
| HIV | 7p13 | 159 | 75.71 | 82.92 |
| HIV | 8q24.3 | 533 | 82.46 | 92.23 |
| HIV | 9q34.11 | 255 | 79.69 | 88.40 |
| HIV | 9q34.13 | 141 | 74.21 | 80.84 |
| HIV | 11q12.3 | 145.5 | 85.59 | 96.55 |
| HIV | 11q13.1 | 484.5 | 193.80 | 246.00 |
| HIV | 11q13.2 | 364 | 145.60 | 179.43 |
| HIV | 12q13.12 | 198 | 82.50 | 92.29 |
| HIV | 12q13.3 | 138 | 92.00 | 105.41 |
| HIV | 15q15.2 | 59 | 73.75 | 80.20 |
| HIV | 16p13.3 | 1015 | 128.48 | 155.79 |
| HIV | 16q22.1 | 448 | 109.27 | 129.26 |
| HIV | 16q24.3 | 322 | 194.59 | 247.09 |
| HIV | 17p13.1 | 349 | 83.10 | 93.11 |
| HIV | 17p13.2 | 346 | 108.13 | 127.68 |
| HIV | 17q21.1 | 38 | 126.67 | 153.28 |
| HIV | 17q25.1 | 295 | 75.64 | 82.82 |
| HIV | 17q25.3 | 805 | 136.55 | 166.93 |
| HIV | 19p13.3 | 631 | 91.45 | 104.65 |
| HIV | 19q13.33 | 415 | 122.06 | 146.92 |

**Table S8.** Association between viral integration sites and fragile sites. Only HIV integrates more often than expected in fragile sites. There is no evidence that HPV, HBV or MCPyV preferentially integrate at fragile sites. Binomial test, α < 0.05.

| **Virus** | **Expected Probability of Being in a Fragile Site** | **Observed Probability of Being in a Fragile Site** | **95% CI** | ***p*-Value** |
| --- | --- | --- | --- | --- |
| **HPV** | 0.219 | 0.238 | 0.204–0.274 | **0.296** |
| **HBV** | 0.219 | 0.238 | 0.214–0.262 | **0.119** |
| **MCPyV** | 0.219 | 0.243 | 0.118–0.412 | **0.693** |
| **HIV** | 0.219 | 0.244 | 0.240–0.248 | ***<2.2 × 10*^−16^** |

**Table S11.** Performance of the optimal random forest models on the test sets, averaged over the 10 models built with different background sets, starting from the genomic features most relevant to the virus.

| **Virus_Window** | **Accuracy** | **Sensitivity** | **Specificity** | **Number of Features** |
| --- | --- | --- | --- | --- |
| HPV_100 | 0.56 | 0.46 | 0.66 | 26.1 |
| HPV_GC_100 | 0.55 | 0.44 | 0.66 | 18.9 |
| HPV_500 | 0.60 | 0.60 | 0.60 | 15.8 |
| HPV_GC_500 | 0.57 | 0.55 | 0.59 | 19.1 |
| HPV_1000 | 0.58 | 0.58 | 0.59 | 11.2 |
| HPV_GC_1000 | 0.56 | 0.60 | 0.52 | 14.9 |
| HPV_10000 | 0.60 | 0.64 | 0.56 | 8.5 |
| HPV_GC_10000 | 0.57 | 0.57 | 0.57 | 9.1 |
| HBV_100 | 0.52 | 0.24 | 0.79 | 85.3 |
| HBV_GC_100 | 0.51 | 0.25 | 0.77 | 94.5 |
| HBV_500 | 0.55 | 0.49 | 0.61 | 13.8 |
| HBV_GC_500 | 0.54 | 0.47 | 0.62 | 12 |
| HBV_1000 | 0.57 | 0.53 | 0.61 | 14 |
| HBV_GC_1000 | 0.56 | 0.54 | 0.58 | 10.8 |
| HBV_10000 | 0.57 | 0.56 | 0.57 | 12.4 |
| HBV_GC_10000 | 0.56 | 0.56 | 0.55 | 14 |
| MCPyV_100 | 0.53 | 0.56 | 0.50 | 9.3 |
| MCPyV_GC_100 | 0.48 | 0.43 | 0.54 | 15.6 |
| MCPyV_500 | 0.49 | 0.48 | 0.50 | 15.4 |
| MCPyV_GC_500 | 0.38 | 0.39 | 0.38 | 16.5 |
| MCPyV_1000 | 0.46 | 0.41 | 0.51 | 7.8 |
| MCPyV_GC_1000 | 0.46 | 0.48 | 0.44 | 22.5 |
| MCPyV_10000 | 0.46 | 0.45 | 0.46 | 15.8 |
| MCPyV_GC_10000 | 0.32 | 0.34 | 0.30 | 31.4 |

**Table S12.** Primers used for ChIP-qPCR.

| **Primer** | **Sequence (5′-3′)** | **Annealing Temperature** |
| --- | --- | --- |
| SAT2-F | Simple ChIP Human Sat2 Repeat Element, Cell Signaling Technology, Category # 5077 | 53 °C |
| SAT2-R | Simple ChIP Human Sat2 Repeat Element, Cell Signaling Technology, Category # 5077 | 53 °C |
| 5′-300-F | CCACAAGACGTGGCATCCT | 53 °C |
| 5′-300-R | CTGCTTCTGTGACTAGTGGTGG | 53 °C |
| 5′J-100-F | CCACCACTAGTCACAGAAGCAG | 53 °C |
| 5′J-100-R | AAAAGTGGACATTACAAGACGTTAG | 53 °C |
| 3′+580-F | TCTCCTCCGAATATGGGAATGAA | 60 °C |
| 3′+580-R | GACACTGGGGATGGAAGTAGC | 60 °C |


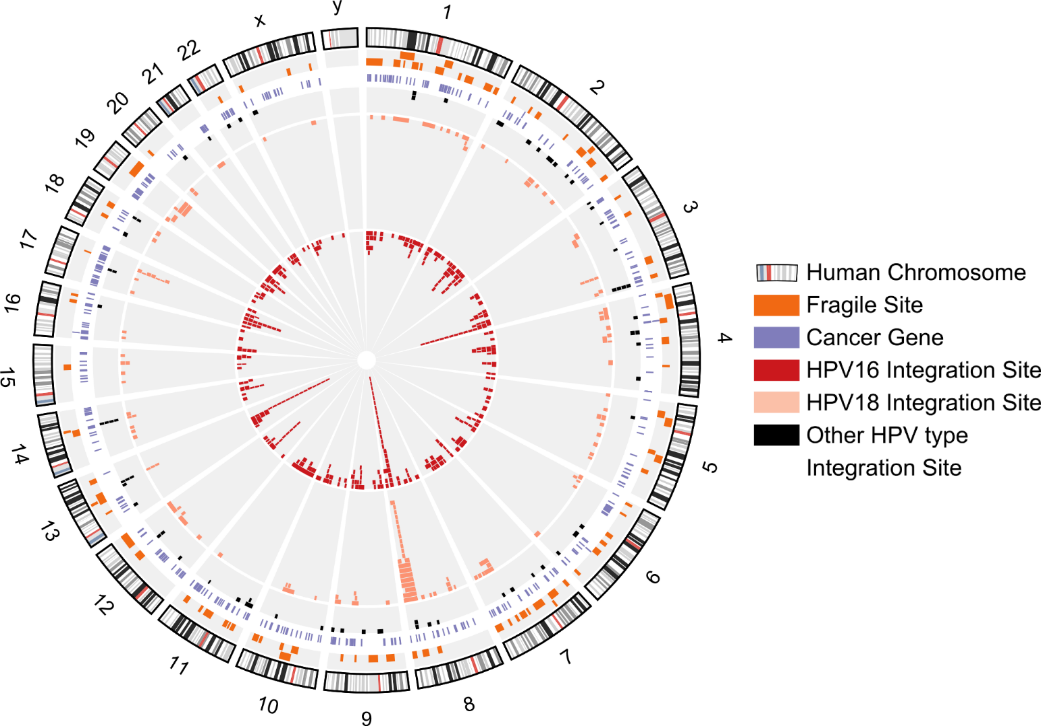


**Figure S1.** Location of HPV integration sites in the human genome. Human chromosomes (1–22, X, Y) are arranged around the circle. The three inner-most rings show the locations of HPV-16, HPV-18 and other HPV types, with multiple events occurring at the same location stacked.


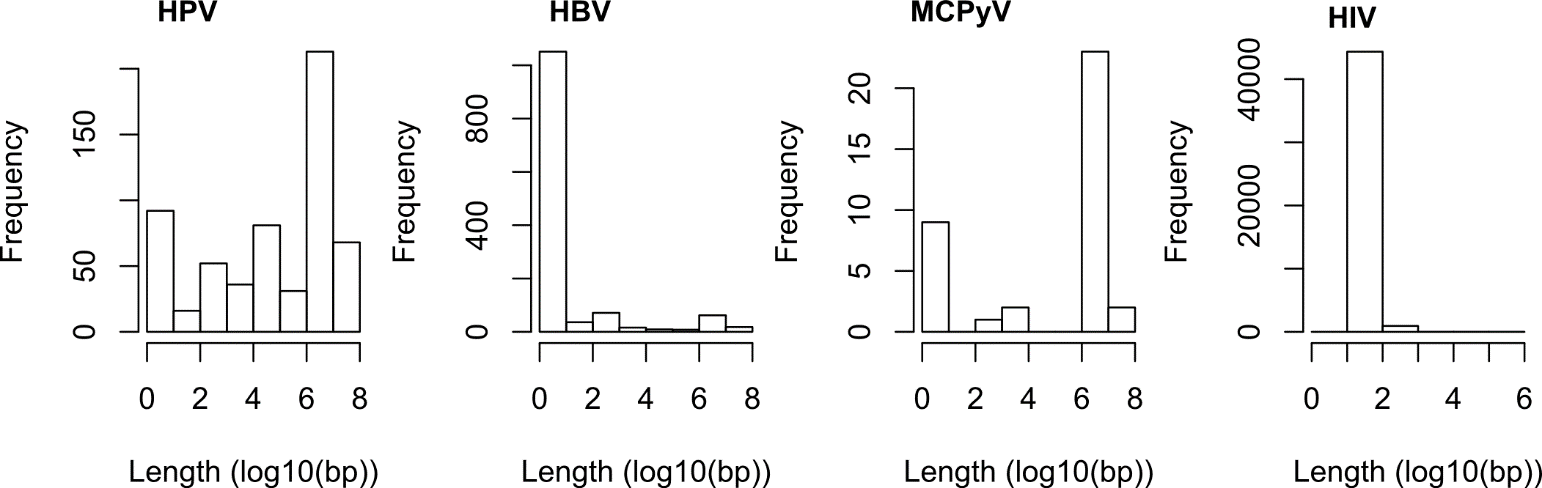


**Figure S2.** Distribution of integration site lengths for each virus.


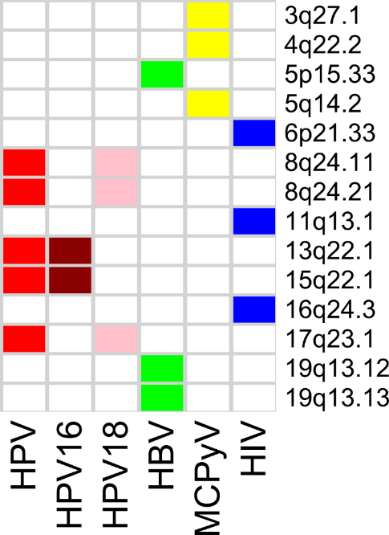


**Figure S3.** Viral integration hotspots at a 99.5 percentile threshold. Novel hotspots were found for HPV (three) and HBV (two). The number of integrations in each cytoband was normalized by the length of the cytoband. Cytobands with a z-score equaling or exceeding the top 0.5% of z-scores for HPV integration were considered hotspots (z ≥ 210.3). * Cytobands suggested to be hotspots of MCPyV integration due to the small number of known integrations.


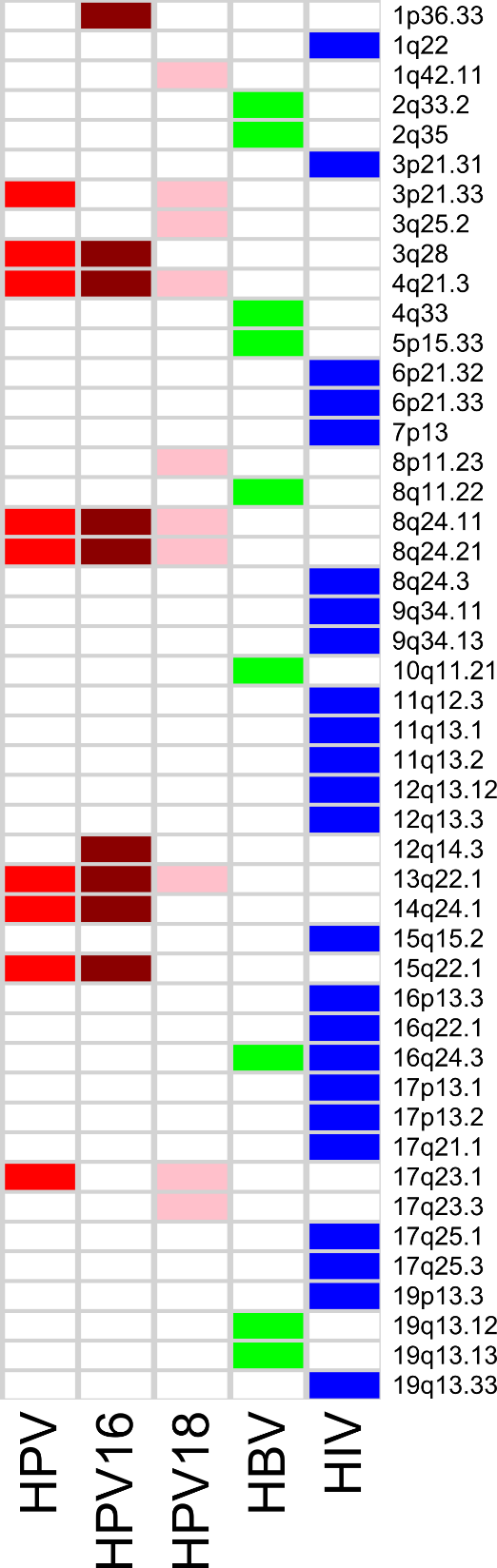


**Figure S4.** Viral integration hotspots at a 99 percentile threshold. The number of integrations in each cytoband was normalized by the length of the cytoband. Cytobands with a z-score equaling or exceeding the top 1% of z-scores for HPV integration were considered hotspots (z ≥ 79.0). MCPyV is not shown because the sample size is too low for the accurate determination of hotspots.





**Figure S5.** Significant differences were detected between viral integration sites and random sites.
All features were considered for each virus. Significance was determined using a two-sided Mann–Whitney U-test with Bonferroni correction, α < 0.05. Comparisons using the gene constraint set are indicated with GC. No significant differences were found for MCPyV.

| **Feature Subsets** | **HPV (%)** | **HBV (%)** | **HIV (%)** |
| --- | --- | --- | --- |
| Same in all cells | 20.90 | 27.13 | 31.49 |
| Relevant cell lines | 54.34 | 43.49 | 36.70 |
| Non-relevant cell lines | 24.75 | 29.39 | 31.82 |


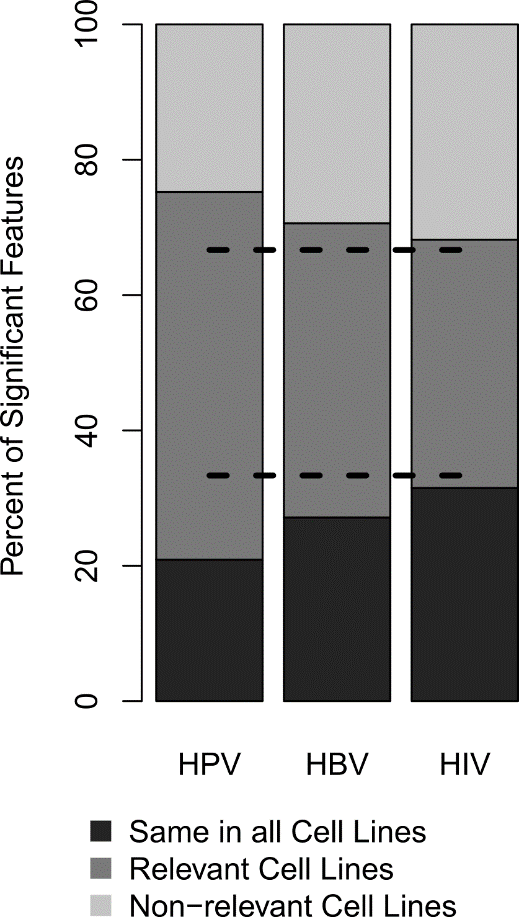


**Figure S6.** Tropism of features significantly associated with integration. Features were divided into three groups: features that are the same in all cells (gene presence and repeats), and those that are from virus-relevant (HPV: HeLa-S3, NHEK, SiHa; HBV: HepG2; HIV: GM12878) or non-relevant cell types. For each virus, the number of features in each group that significantly differed between integration and random sites at any window size and with either background set (Supplementary Figure S5) were summed and normalized by the total number of features in that group and presented as a percent of the total. If there was no effect of cell type, all three groups would have 33.33% of the total. The dashed lines are at 33.33% and 66.66%. HPV and HBV have a higher percentage of significant features in the cell lines most relevant to these viruses, indicating cell type specificity of features near integration sites.


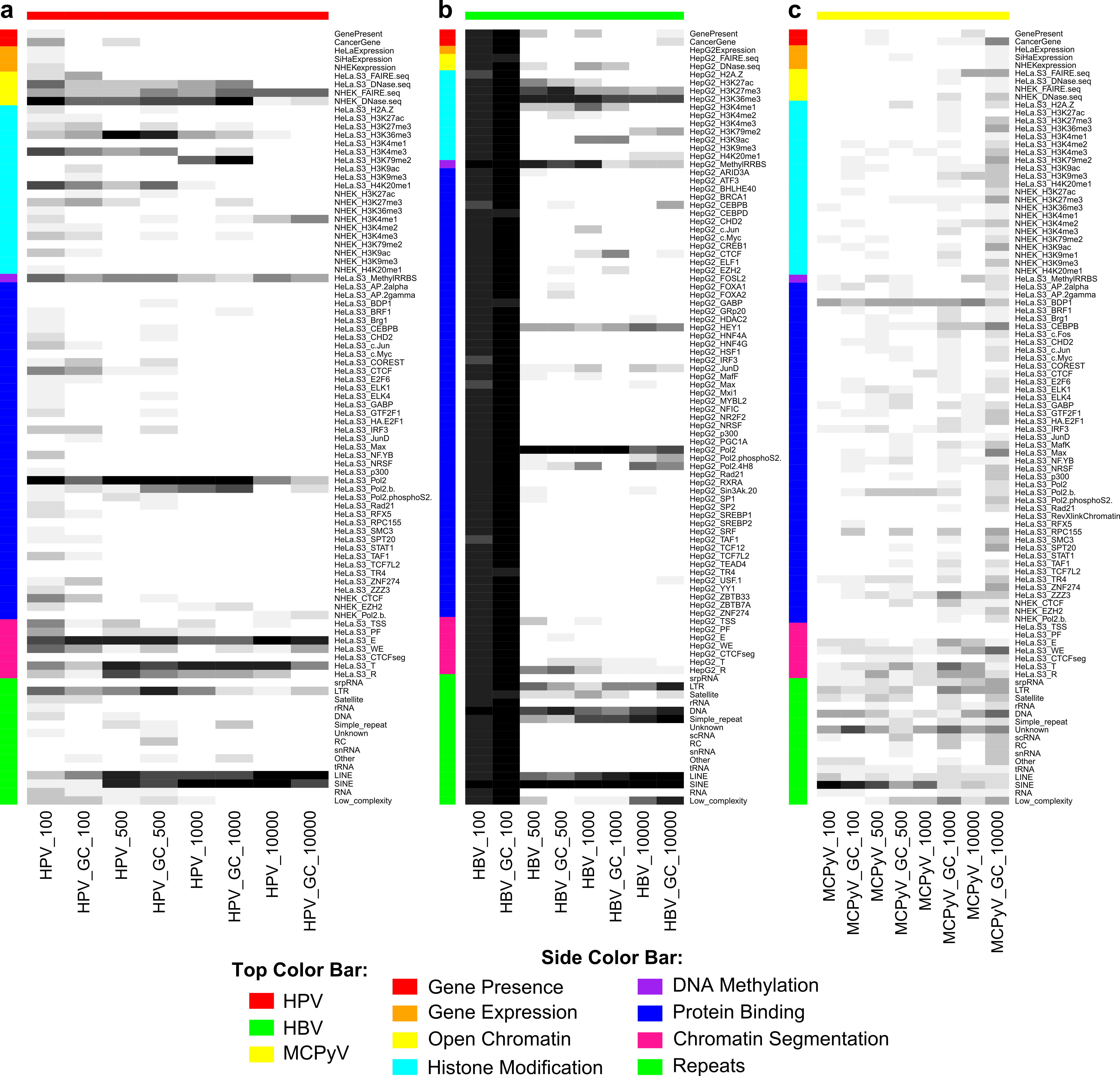


**Figure S7.** Predictive genomic features for each DNA tumor virus. Random forest models were developed for each virus and window size, using either the background set or the gene constraint set as the negative class. Starting from only the genomic features that were considered relevant to each virus, feature elimination was used to select the smallest set of features that gave an ROC within 2% of the best model using three-fold cross-validation repeated 10 times on the training set. The optimal model was then used to classify a held-out test set (75% of data for training, 25% for testing). The entire process was repeated 10 times, once for each of the randomly-selected background sets. The number of times each feature was selected for inclusion in the optimal model is shown (white: zero, black: 10). Only features selected at least once are shown. (**a**) Features predictive of HPV integration. (**b**) Features predictive of HPV integration. (**c**) Features predictive of HPV integration. Comparisons using the gene constraint set are indicated with GC.

References

1. Thorland, E.C.; Myers, S.L.; Persing, D.H.; Sarkar, G.; McGovern, R.M.; Gostout, B.S.; Smith, D.I. Human papillomavirus type 16 integrations in cervical tumors frequently occur in common fragile sites. *Cancer Res.* **2000**, *60*, 5916–5921.
2. Schmitz, M.; Driesch, C.; Jansen, L.; Runnebaum, I.B.; Dürst, M. Non-random integration of the HPV genome in cervical cancer. *PLoS ONE* **2012**, *7*, e39632, doi:10.1371/journal.pone.0039632.
3. Annunziata, C.; Buonaguro, L.; Buonaguro, F.M.; Tornesello, M.L. Characterization of the human papillomavirus (HPV) integration sites into genital cancers. *Pathol. Oncol. Res.* **2012**, *18*, 803–808.
4. Dall, K.L.; Scarpini, C.G.; Roberts, I.; Winder, D.M.; Stanley, M.A.; Muralidhar, B.; Herdman, M.T.; Pett, M.R.; Coleman, N. Characterization of naturally occurring HPV16 integration sites isolated from cervical keratinocytes under noncompetitive conditions. *Cancer Res.* **2008**, *68*, 8249–8259.
5. Couturier, J.; Sastre-Garau, X.; Schneider-Maunoury, S.; Labib, A.; Orth, G. Integration of papillomavirus DNA near myc genes in genital carcinomas and its consequences for proto-oncogene expression. *J. Virol.* **1991**, *65*, 4534–4538.
6. Das, P.; Thomas, A.; Mahantshetty, U.; Shrivastava, S.K.; Deodhar, K.; Mulherkar, R. HPV genotyping and site of viral integration in cervical cancers in Indian women. *PLoS ONE* **2012**, *7*, e41012, doi:10.1371/journal.pone.0041012.
7. Lace, M.J.; Anson, J.R.; Klussmann, J.P.; Wang, D.H.; Smith, E.M.; Haugen, T.H.; Turek, L.P. Human papillomavirus type 16 (HPV-16) genomes integrated in head and neck cancers and in HPV-16-immortalized human keratinocyte clones express chimeric virus-cell mRNAs similar to those found in cervical cancers. *J. Virol.* **2011**, *85*, 1645–1654.
8. Tang, K.-W.; Alaei-Mahabadi, B.; Samuelsson, T.; Lindh, M.; Larsson, E. The landscape of viral expression and host gene fusion and adaptation in human cancer. *Nat. Commun.* **2013**, *4*, 1–9.
9. Ferber, M.J.; Montoya, D.P.; Yu, C.; Aderca, I.; McGee, A.; Thorland, E.C.; Nagorney, D.M.; Gostout, B.S.; Burgart, L.J.; Boix, L.; *et al*. Integrations of the hepatitis B virus (HBV) and human papillomavirus (HPV) into the human telomerase reverse transcriptase (hTERT) gene in liver and cervical cancers. *Oncogene* **2003**, *22*, 3813–3820.
10. Kraus, I.; Driesch, C.; Vinokurova, S.; Hovig, E.; Schneider, A.; von Knebel Doeberitz, M.; Dürst, M. The majority of viral-cellular fusion transcripts in cervical carcinomas cotranscribe cellular sequences of known or predicted genes. *Cancer Res.* **2008**, *68*, 2514–2522.
11. Ziegert, C.; Wentzensen, N.; Vinokurova, S.; Kisseljov, F.; Einenkel, J.; Hoeckel, M.; von Knebel Doeberitz, M. A comprehensive analysis of HPV integration loci in anogenital lesions combining transcript and genome-based amplification techniques. *Oncogene* **2003**, *22*, 3977–3984.
12. Wentzensen, N.; Ridder, R.; Klaes, R. Characterization of viral-cellular fusion transcripts in a large series of HPV16 and 18 positive anogenital lesions. *Oncogene* **2002**, *21*, 419–426.
13. Winder, D.; Pett, M.; Foster, N.; Shivji, M.; Herdman, M.; Stanley, M.; Venkitaraman, A.; Coleman, N. An increase in DNA double-strand breaks, induced by Ku70 depletion, is associated with human papillomavirus 16 episome loss and de novo viral integration events. *J. Pathol.* **2007**, *213*, 27–34.
14. Sastre-Garau, X.; Schneider-Maunoury, S.; Couturier, J.; Orth, G. Human papillomavirus type 16 DNA is integrated into chromosome region 12q14-q15 in a cell line derived from a vulvar intraepithelial neoplasia. *Cancer Genet. Cytogenet.* **1990**, *44*, 243–251.
15. Kalantari, M.; Blennow, E.; Hagmar, B.; Johansson, B. Physical state of HPV16 and chromosomal mapping of the integrated form in cervical carcinomas. *Diagn. Mol. Pathol.* **2001**, *10*, 46–54.
16. Smith, P.; Friedman, C.; Bryant, E.; McDougall, J. Viral integration and fragile sites in human papillomavirus‐lmmortalized human keratinocyte cell lines. *Genes Chromosomes Cancer* **1992**, *5*, 150–157.
17. Klimov, E.; Vinokourova, S.; Moisjak, E.; Rakhmanaliev, E.; Kobseva, V.; Laimins, L.; Kisseljov, F.; Sulimova, G. Human papilloma viruses and cervical tumours: Mapping of integration sites and analysis of adjacent cellular sequences. *BMC Cancer* **2002**, *2*, 24, doi:10.1186/1471-2407-2-24.
18. Ferber, M.J.; Thorland, E.C.; Brink, A.A.T.P.; Rapp, A.K.; Phillips, L.A.; McGovern, R.; Gostout, B.S.; Cheung, T.H.; Chung, T.K.H.; Fu, W.Y.; *et al*. Preferential integration of human papillomavirus type 18 near the c-myc locus in cervical carcinoma. *Oncogene* **2003**, *22*, 7233–7242.
19. Ojesina, A.I.; Lichtenstein, L.; Freeman, S.S.; Pedamallu, C.S.; Imaz-Rosshandler, I.; Pugh, T.J.; Cherniack, A.D.; Ambrogio, L.; Cibulskis, K.; Bertelsen, B.; *et al*. Landscape of genomic alterations in cervical carcinomas. *Nature* **2014**, *506*, 371–375.
20. Akagi, K.; Li, J.; Broutian, T.R.; Padilla-Nash, H.; Xiao, W.; Jiang, B.; Rocco, J.W.; Teknos, T.N.; Kumar, B.; Wangsa, D.; *et al*. Genome-wide analysis of HPV integration in human cancers reveals recurrent, focal genomic instability. *Genome Res.* **2014**, *24*, 185–199.
21. Landry, J.J.M.; Pyl, P.T.; Rausch, T.; Zichner, T.; Tekkedil, M.M.; Stütz, A.M.; Jauch, A.; Aiyar, R.S.; Pau, G.; Delhomme, N.; *et al*. The Genomic and Transcriptomic Landscape of a HeLa Cell Line. *G3 (Bethesda)* **2013**, *3*, 1213–1224.
22. Gao, G.; Johnson, S.H.; Kasperbauer, J.L.; Eckloff, B.W.; Tombers, N.M.; Vasmatzis, G.; Smith, D.I. Mate pair sequencing of oropharyngeal squamous cell carcinomas reveals that HPV integration occurs much less frequently than in cervical cancer. *J. Clin. Virol.* **2014**, *59*, 195–200.
23. Einstein, M.; Cruz, Y.; El-Awady, M.; Popescu, N.C.; DiPaolo, J.A.; van Ranst, M.; Kadish, A.S.; Romney, S.; Runowicz, C.D.; Burk, R.D. Human genome sequence localizes human papillomavirus type 16 DNA integrated into the TNFAIP2 gene in a fatal cervical cancer from a 39-year-old woman. *Clin. Cancer Res.* **2002**, *8*, 549–554.
24. Luft, F.; Klaes, R.; Nees, M.; Dürst, M.; Heilmann, V.; Melsheimer, P.; von Knebel Doeberitz, M. Detection of integrated papillomavirus sequences by ligation-mediated PCR (DIPS-PCR) and molecular characterization in cervical cancer cells. *Int. J. Cancer* **2001**, *92*, 9–17.
25. Shera, K.A.; Shera, C.A.; McDougall, J.K. Small tumor virus genomes are integrated near nuclear matrix attachment regions in transformed cells. *J. Virol.* **2001**, *75*, 12339–12346.
26. Murakami, Y.; Saigo, K.; Takashima, H.; Minami, M.; Okanoue, T.; Bréchot, C.; Paterlini-Bréchot, P. Large scaled analysis of hepatitis B virus (HBV) DNA integration in HBV related hepatocellular carcinomas. *Gut* **2005**, *54*, 1162–1168.
27. Sung, W.-K.; Zheng, H.; Li, S.; Chen, R.; Liu, X.; Li, Y.; Lee, N.P.; Lee, W.H.; Ariyaratne, P.N.; Tennakoon, C.; *et al*. Genome-wide survey of recurrent HBV integration in hepatocellular carcinoma. *Nat. Genet.* **2012**, *44*, 765–769.
28. Gozuacik, D.; Murakami, Y.; Saigo, K.; Chami, M.; Mugnier, C.; Lagorce, D.; Okanoue, T.; Urashima, T.; Bréchot, C.; Paterlini-Bréchot, P. Identification of human cancer-related genes by naturally occurring Hepatitis B Virus DNA tagging. *Oncogene* **2001**, *20*, 6233–6240.
29. Paterlini-Bréchot, P.; Saigo, K.; Murakami, Y.; Chami, M.; Gozuacik, D.; Mugnier, C.; Lagorce, D.; Bréchot, C. Hepatitis B virus-related insertional mutagenesis occurs frequently in human liver cancers and recurrently targets human telomerase gene. *Oncogene* **2003**, *22*, 3911–3916.
30. Saigo, K.; Yoshida, K.; Ikeda, R.; Sakamoto, Y.; Murakami, Y.; Urashima, T.; Asano, T.; Kenmochi, T.; Inoue, I. Integration of hepatitis B virus DNA into the myeloid/lymphoid or mixed-lineage leukemia (MLL4) gene and rearrangements of MLL4 in human hepatocellular carcinoma. *Hum. Mutat.* **2008**, *29*, 703–708.
31. Bonilla Guerrero, R.; Roberts, L.R. The role of hepatitis B virus integrations in the pathogenesis of human hepatocellular carcinoma. *J. Hepatol.* **2005**, *42*, 760–777.
32. Koshy, R.; Koch, S.; Freytag von Loringhoven, A.; Kahmann, R.; Murray, K.; Hofschneider, P.H. Integration of hepatitis B virus DNA: Evidence for integration in the single-stranded gap. *Cell* **1983**, *34*, 215–223.
33. Ziemer, M.; Garcia, P.; Shaul, Y.; Rutter, W.J. Sequence of hepatitis B virus DNA incorporated into the genome of a human hepatoma cell line. *J. Virol.* **1985**, *53*, 885–892.
34. Graef, E.; Caselmann, W.H.; Wells, J.; Koshy, R. Insertional activation of mevalonate kinase by hepatitis B virus DNA in a human hepatoma cell line. *Oncogene* **1994**, *9*, 81–87.
35. Yaginuma, K.; Kobayashi, M.; Yoshida, E.; Koike, K. Hepatitis B virus integration in hepatocellular carcinoma DNA: Duplication of cellular flanking sequences at the integration site. *Proc. Natl. Acad. Sci. USA* **1985**, *82*, 4458–4462.
36. Chen, J.Y.; Harrison, T.J.; Tsuei, D.J.; Hsu, T.Y.; Zuckerman, A.J.; Chan, T.S.; Yang, C.S. Analysis of integrated hepatitis B virus DNA and flanking cellular sequences in the hepatocellular carcinoma cell line HCC36. *Intervirology* **1994**, *37*, 41–46.
37. Horikawa, I.; Barrett, J.C. cis-Activation of the Human Telomerase Gene (hTERT) by the Hepatitis B Virus Genome. *JNCI J. Natl. Cancer Inst.* **2001**, *93*, 1171–1173.
38. Yaginuma, K.; Kobayashi, H.; Kobayashi, M.; Morishima, T.; Matsuyama, K.; Koike, K. Multiple integration site of hepatitis B virus DNA in hepatocellular carcinoma and chronic active hepatitis tissues from children. *J. Virol.* **1987**, *61*, 1808–1813.
39. Pineau, P.; Marchio, A.; Mattei, M.G.; Kim, W.H.; Youn, J.K.; Tiollais, P.; Dejean, A. Extensive analysis of duplicated-inverted hepatitis B virus integrations in human hepatocellular carcinoma. *J. Gen. Virol.* **1998**, *79*, 591–600.
40. Dejean, A.; Sonigo, P.; Wain-Hobson, S.; Tiollais, P. Specific hepatitis B virus integration in hepatocellular carcinoma DNA through a viral 11-base-pair direct repeat. *Proc. Natl. Acad. Sci. USA* **1984**, *81*, 5350–5354.
41. Shih, C.; Burke, K.; Chou, M.J.; Zeldis, J.B.; Yang, C.S.; Lee, C.S.; Isselbacher, K.J.; Wands, J.R.; Goodman, H.M. Tight clustering of human hepatitis B virus integration sites in hepatomas near a triple-stranded region. *J. Virol.* **1987**, *61*, 3491–3498.
42. Hatada, I.; Tokino, T.; Ochiya, T.; Matsubara, K. Co-amplification of integrated hepatitis B virus DNA and transforming gene hst-1 in a hepatocellular carcinoma. *Oncogene* **1988**, *3*, 537–540.
43. Zhou, Y.Z.; Slagle, B.L.; Donehower, L.A.; vanTuinen, P.; Ledbetter, D.H.; Butel, J.S. Structural analysis of a hepatitis B virus genome integrated into chromosome 17p of a human hepatocellular carcinoma. *J. Virol.* **1988**, *62*, 4224–4231.
44. Hino, O.; Ohtake, K.; Rogler, C.E. Features of two hepatitis B virus (HBV) DNA integrations suggest mechanisms of HBV integration. *J. Virol.* **1989**, *63*, 2638–2643.
45. Wang, J.; Chenivesse, X.; Henglein, B.; Bréchot, C. Hepatitis B virus integration in a cyclin A gene in a hepatocellular carcinoma. *Nature* **1990**, *343*, 555–557.
46. Quade, K.; Saldanha, J.; Thomas, H.; Monjardino, J. Integration of hepatitis B virus DNA through a mutational hot spot within the cohesive region in a case of hepatocellular carcinoma. *J. Gen. Virol.* **1992**, *73*, 179–182.
47. Zhang, X.K.; Egan, J.O.; Huang, D.; Sun, Z.L.; Chien, V.K.; Chiu, J.F. Hepatitis B virus DNA integration and expression of an erb B-like gene in human hepatocellular carcinoma. *Biochem. Biophys. Res. Commun.* **1992**, *188*, 344–351.
48. Tsuei, D.-J.; Hsu, T.-Y.; Chen, J.-Y.; Chang, M.-H.; Hsu, H.-C.; Yang, C.-S. Analysis of integrated hepatitis B virus DNA and flanking cellular sequences in a childhood hepatocellular carcinoma. *J. Med. Virol.* **1994**, *42*, 287–293.
49. Chami, M.; Gozuacik, D.; Saigo, K.; Capiod, T.; Falson, P.; Lecoeur, H.; Urashima, T.; Beckmann, J.; Gougeon, M.L.; Claret, M.; *et al*. Hepatitis B virus-related insertional mutagenesis implicates SERCA1 gene in the control of apoptosis. *Oncogene* **2000**, *19*, 2877–2886.
50. Tsuei, D.-J.; Chang, M.-H.; Chen, P.-J.; Hsu, T.-Y.; Ni, Y.-H. Characterization of integration patterns and flanking cellular sequences of hepatitis B virus in childhood hepatocellular carcinomas. *J. Med. Virol.* **2002**, *68*, 513–521.
51. Nagaya, T.; Nakamura, T.; Tokino, T.; Tsurimoto, T.; Imai, M.; Mayumi, T.; Kamino, K.; Yamamura, K.; Matsubara, K. The mode of hepatitis B virus DNA integration in chromosomes of human hepatocellular carcinoma. *Genes Dev.* **1987**, *1*, 773–782.
52. Ding, D.; Lou, X.; Hua, D.; Yu, W.; Li, L.; Wang, J.; Gao, F.; Zhao, N.; Ren, G.; Li, L.; *et al*. Recurrent targeted genes of hepatitis B virus in the liver cancer genomes identified by a next-generation sequencing-based approach. *PLoS Genet.* **2012**, *8*, e1003065, doi:10.1371/journal.pgen.1003065.
53. Jiang, S.; Yang, Z.; Li, W.; Li, X.; Wang, Y.; Zhang, J.; Xu, C.; Chen, P.-J.; Hou, J.; McCrae, M.A.; *et al*.
    Re-evaluation of the carcinogenic significance of hepatitis B virus integration in hepatocarcinogenesis. *PLoS ONE* **2012**, *7*, e40363, doi:10.1371/journal.pone.0040363.
54. Jiang, Z.; Jhunjhunwala, S.; Liu, J.; Haverty, P.M.; Kennemer, M.I.; Guan, Y.; Lee, W.; Carnevali, P.; Stinson, J.; Johnson, S.; *et al*. The effects of hepatitis B virus integration into the genomes of hepatocellular carcinoma patients. *Genome Res.* **2012**, *22*, 593–601.
55. Lau, C.-C.; Sun, T.; Ching, A.K.K.; He, M.; Li, J.-W.; Wong, A.M.; Co, N.N.; Chan, A.W.H.; Li, P.-S.; Lung, R.W.M.; *et al*. Viral-human chimeric transcript predisposes risk to liver cancer development and progression. *Cancer Cell* **2014**, *25*, 335–349.
56. Fujimoto, A.; Totoki, Y.; Abe, T.; Boroevich, K.A.; Hosoda, F.; Nguyen, H.H.; Aoki, M.; Hosono, N.; Kubo, M.; Miya, F.; *et al*. Whole-genome sequencing of liver cancers identifies etiological influences on mutation patterns and recurrent mutations in chromatin regulators. *Nat. Genet.* **2012**, *44*, 760–764.
57. Mason, W.S.; Liu, C.; Aldrich, C.E.; Litwin, S.; Yeh, M.M. Clonal expansion of normal-appearing human hepatocytes during chronic hepatitis B virus infection. *J. Virol.* **2010**, *84*, 8308–8015.
58. Tamori, A.; Yamanishi, Y.; Kawashima, S.; Kanehisa, M.; Enomoto, M.; Tanaka, H.; Kubo, S.; Shiomi, S.; Nishiguchi, S. Alteration of gene expression in human hepatocellular carcinoma with integrated hepatitis B virus DNA. *Clin. Cancer Res.* **2005**, *11*, 5821–5826.
59. Wang, Y.; Lau, S.H.; Sham, J.S.-T.; Wu, M.-C.; Wang, T.; Guan, X.-Y. Characterization of HBV integrants in 14 hepatocellular carcinomas: Association of truncated X gene and hepatocellular carcinogenesis. *Oncogene* **2004**, *23*, 142–148.
60. Duncavage, E.J.; Magrini, V.; Becker, N.; Armstrong, J.R.; Demeter, R.T.; Wylie, T.; Abel, H.J.; Pfeifer, J.D. Hybrid capture and next-generation sequencing identify viral integration sites from formalin-fixed, paraffin-embedded tissue. *J. Mol. Diagn.* **2011**, *13*, 325–333.
61. Feng, H.; Shuda, M.; Chang, Y.; Moore, P. Clonal integration of a polyomavirus in human Merkel cell carcinoma. *Science* **2008**, *319*, 1096–1100.
62. Guastafierro, A.; Feng, H.; Thant, M. Characterization of an early passage Merkel cell polyomavirus-positive Merkel cell carcinoma cell line, MS-1, and its growth in NOD scid gamma mice. *J. Virol. Methods* **2013**, *187*, 6–14.
63. Sastre-Garau, X.; Peter, M.; Avril, M.-F.; Laude, H.; Couturier, J.; Rozenberg, F.; Almeida, A.; Boitier, F.; Carlotti, A.; Couturaud, B.; *et al*. Merkel cell carcinoma of the skin: Pathological and molecular evidence for a causative role of MCV in oncogenesis. *J. Pathol.* **2009**, *218*, 48–56.
64. Laude, H.C.; Jonchère, B.; Maubec, E.; Carlotti, A.; Marinho, E.; Couturaud, B.; Peter, M.; Sastre-Garau, X.; Avril, M.-F.; Dupin, N.; *et al*. Distinct merkel cell polyomavirus molecular features in tumour and non tumour specimens from patients with merkel cell carcinoma. *PLoS Pathog.* **2010**, *6*, e1001076, doi:10.1371/journal.ppat.1001076.
65. Martel-Jantin, C.; Filippone, C.; Cassar, O.; Peter, M.; Tomasic, G.; Vielh, P.; Brière, J.; Petrella, T.; Aubriot-Lorton, M.H.; Mortier, L.; *et al*. Genetic variability and integration of Merkel cell polyomavirus in Merkel cell carcinoma. *Virology* **2012**, *426*, 134–142.
66. Hashida, Y.; Imajoh, M.; Nemoto, Y.; Kamioka, M.; Taniguchi, A.; Taguchi, T.; Kume, M.; Orihashi, K.; Daibata, M. Detection of Merkel cell polyomavirus with a tumour-specific signature in non-small cell lung cancer. *Br. J. Cancer* **2013**, *108*, 629–637.
67. Wang, G.P.; Ciuffi, A.; Leipzig, J.; Berry, C.C.; Bushman, F.D. HIV integration site selection: Analysis by massively parallel pyrosequencing reveals association with epigenetic modifications. *Genome Res.* **2007**, *17*, 1186–1194.

© 2015 by the authors; licensee MDPI, Basel, Switzerland. This article is an open access article distributed under the terms and conditions of the Creative Commons by Attribution (CC-BY) license (http://creativecommons.org/licenses/by/4.0/).
